# An RNAi screen of Rab GTPase genes in *Caenorhabditis elegans* reveals that morphogenesis has a higher demand than stem cell niche maintenance for *rab-1* in the somatic cells of the reproductive system

**DOI:** 10.1093/g3journal/jkaf085

**Published:** 2025-04-16

**Authors:** Noor Singh, Kayt Scott, Jayce Proctor, Kacy Lynn Gordon

**Affiliations:** Department of Biology, The University of North Carolina at Chapel Hill, Chapel Hill, NC 27599, USA; Department of Biology, The University of North Carolina at Chapel Hill, Chapel Hill, NC 27599, USA; Department of Biology, The University of North Carolina at Chapel Hill, Chapel Hill, NC 27599, USA; Department of Biology, The University of North Carolina at Chapel Hill, Chapel Hill, NC 27599, USA; UNC Lineberger Comprehensive Cancer Center, The University of North Carolina at Chapel Hill, Chapel Hill, NC 27599, USA

**Keywords:** Rab GTPase, RNAi, *C. elegans*, gonad, germline, stem cell, gamete differentiation, Animalia

## Abstract

Membrane trafficking is a crucial function of all cells and is regulated at multiple levels from vesicle formation, packaging, and localization to fusion, exocytosis, and endocytosis. Rab GTPase proteins are core regulators of eukaryotic membrane trafficking, but developmental roles of specific Rab GTPases are less well characterized, potentially because of their essentiality for basic cellular function. *Caenorhabditis elegans* gonad development entails the coordination of cell growth, proliferation, and migration—processes in which membrane trafficking is known to be required. Here, we take an organ-focused approach to Rab GTPase function in vivo to assess the roles of Rab genes in reproductive system development. We performed a whole-body RNAi screen of the entire Rab family in *C. elegans* to uncover Rabs essential for gonad development. Notable gonad defects resulted from RNAi knockdown of *rab-1*, the key regulator of ER–Golgi trafficking. We then examined the effects of tissue-specific RNAi knockdown of *rab-1* in somatic reproductive system and germline cells. We interrogated the dual functions of the distal tip cell as both a leader cell of gonad organogenesis and the germline stem cell niche. We find that *rab-1* functions cell-autonomously and non-cell-autonomously to regulate both somatic gonad and germline development. Gonad migration, elongation, and gamete differentiation—but surprisingly not germline stem niche function—are highly sensitive to *rab-1* RNAi.

## Introduction

Members of the Rab family of GTPases—within the Ras superfamily—broadly regulate vesicular traffic within eukaryotic cells, including in specialized cell functions such as cell division (Gibieža and Prekeris 2018), cell polarity (Parker *et al*. 2018), and the release of neuropeptides into the synapse (Sasidharan *et al*. 2012). When systematically knocked out in a cell culture system with a method that is sensitive to potential redundancy among paralogs, specific functions for Rab family members have been revealed (Homma *et al*. 2019). While Rab GTPase-dependent membrane trafficking has been studied in *Caenorhabditis elegans* (Sato *et al*. 2014), only a handful of the 31 members of the Rab family have been investigated at a developmental genetic level (Lundquist 2006; Ghosh and Sternberg 2014). We hypothesize that the absence of literature exploring the roles of many Rab genes in *C. elegans* development is due to both redundancy among members of this family and essentiality of many Rab family members; nearly half of the Rab genes have been shown to cause lethality with strong loss of function in *C. elegans* (Table 1).

**Table 1.** RNAi screen of all identified *C. elegans* rab GTPase genes in a strain with markers for the germline and somatic gonad.

*C. elegans* gene	Clone number	Human ortholog (Gallegos *et al*. 2012)	RNAi exposure timing	Most severe known *C. elegans* LOF phenotype	Reference	Gonad defect observed	Severity (mild, moderate, severe)
*rab-1*	C39F7.4	RAB1A	Maternal, L1	Embryonic lethal	Couillault *et al*. (2004)	Yes	Severe
*unc-108/rab-2*	F53F10.4	RAB2B	Maternal	Embryonic lethal	Li *et al*. (2009)	No	
*rab-3*	C18A3.6	RAB3	L1	Behavioral defect	Gracheva *et al*. (2008); Nonet *et al*. (1997)	No	
*rab-5*	F26H9.6	RAB5C	Maternal	L1 lethal	Huang *et al*. (2022)	Yes	Moderate
*rab-6.1*	F59B2.7	RAB6A	L1	Embryonic lethal (when codepleted with *rab-6.2*)	Audhya *et al*. (2007); Zhang *et al*. (2008)	No	
*rab-6.2*	T25G12.4	RAB6A	L1	DTC migration defect (see *rab-6.1*)	Shafaq-Zadah *et al*. (2015)	Yes	Severe
*rab-7*	W03C9.3	RAB7A	Maternal	Embryonic lethal	Nieto *et al*. (2010)	Yes	moderate
*rab-8*	D1037.4	RAB8A	L1	DTC migration defect	Cram *et al*. (2006)	No	
*rab-10*	T23H2.5	RAB10	Maternal	Sterile, sick	Ceron *et al*. (2007)	Yes	Moderate
*rab-11.1*	F53G12.1	RAB11	Maternal	Zygotic lethal	Sato *et al*. (2008)	Yes	Mild
*rab-11.2*	W04G5.2	RAB11	Maternal	Slow growth	Simmer *et al*. (2003)	Yes	Moderate to severe
*rab-14^a^*,*^b^*	K09A9.2	RAB14	Maternal	Accumulated cell corpses	Guo *et al*. (2010)	No	
*rab-18*	Y92C3B.3	RAB18	L1	Lethal	tm2052 2024 (variation)—WormBase	No	
*rab-19*	Y62E10A.9	RAB19	L1	Axonal and dendritic protein sorting	Li *et al*. (2016)	Yes	Severe
*rab-21*	T01B7.3	RAB21	L1	Embryonic lethal	Fernandez *et al*. (2005)	No	
*rab-27/aex-6*	Y87G2A.4	RAB27A	L1	Larval arrest	Mukhopadhyay *et al*. (2007)	No	
*rab-28*	Y11D7A.4	RAB28	Maternal	None		No	
*rab-30*	Y45F3A.2	RAB30	L1	None		No	
*rab-33*	F43D9.2	RAB33A	L1	None		No	
*rab-35*	Y47D3A.25	RAB35	L1	Embryonic lethal	Haley and Zhou (2021)	No	
*rab-37*	W01H2.3	RAB37	Maternal	None		No	
*rab-39*	D2013.1	RAB39	L1	Oxidative stress phenotype	Takenaka *et al*. (2013)	No	
*-*	C56E6.2	No direct ortholog	L1	None		Yes	Severe
*glo-1^b^*	R07B1.12	RAB32	L1	Embryonic lethal	Hermann *et al*. (2005)	Yes	Severe
*rabr-2*	4R79.2	RAB44 related	L1	Synthetic growth and intestinal defects	Sakaki *et al*. (2012)	Yes	Severe
*rabr-4 and rabr-3^c^*	F11A5.3	RAB2 related	L1	Reduced lipid content*^d^*	Ashrafi *et al*. (2003)	No	
*rabr-1*	K02E10.1	No direct ortholog	L1	Dendrite branching defect	Salazar *et al*. (2024)	No	
*tag-312/rsef-1*	C33D12.6	RAB45/RASEF related	L1	utse defect	Ghosh and Sternberg (2014)	No	
*-*	Y71H2AM.12*^b^*	RAB6 related	L1	None		No	
*-*	ZK669.5	RAB23 related	L1	None		No	

^a^The sequenced Ahringer Library clone K09A9.2 (*rab-14)* does not map to the *rab-14* sequence, but instead largely maps only to the empty RNAi vector (L4440).

^b^RNAi clone designed for this study.

^c^clone F11A5.3 mapped to both *rabr-4* and *rabr-3* exons.

^d^ *rabr-4* defect; no documented *rabr-3* defects.

**Fig. 1.**
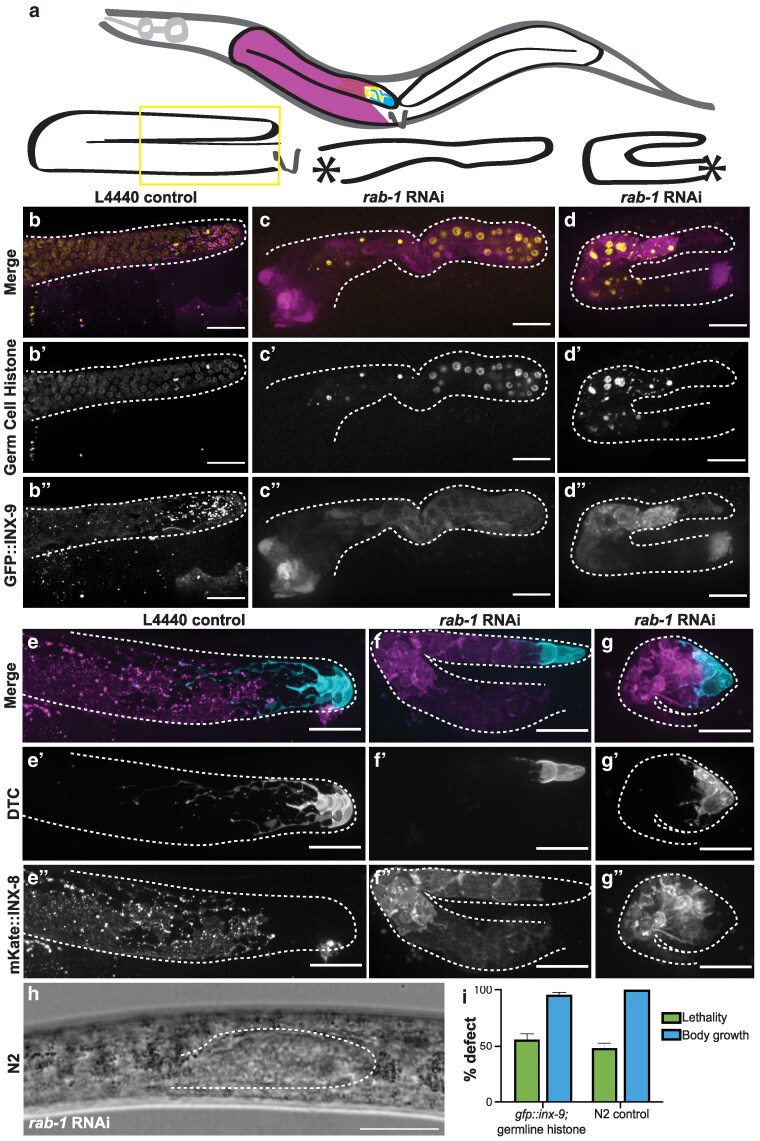
Whole-body *rab-1* knockdown leads to large-scale defects in the gonad. a) Cartoon showing position of 2 gonad arms inside of worm body (above) and representative gonad shapes of control (lower left) and *rab-1* RNAi gonads (center and right). Vulva shown with V, and location where proximal gonad fails to form in RNAi samples shown with asterisks. b)–d) Representative images of germ cells (yellow; *mex-5p::H2B::mCherry::nos-2 3′UTR*) (b′–d′) and somatic gonad DTC and sheath cells (magenta; GFP::INX-9) (b″–d″). Adults that escaped severe embryonic and larval defects after hatching on whole-body *rab-1* RNAi (imaged 4 days post-maternal exposure) (c–c″, d–d″) have fewer germ cells and smaller gonads in *n* = 4/16 worms compared with adults hatched on L4440 empty RNAi vector (b-b″) and display turning and gonad growth defects (c-c″) as well as catastrophic gonad defects (d–d″). e)–g) Representative images of strain expressing markers for the DTC (cyan*; lag-2p::mNeonGreen::PLC^δPH^*) (e′–g′) and gonadal sheath cells (magenta; mKate::INX-8) (e″–g″). Adults that escaped severe embryonic and larval defects after hatching on *rab-1* RNAi (f–f″, g–g″) have DTC and sheath cells in the correct relative positions, but with aberrant morphology in *n* = 6/12 worms compared to L4440 treated controls (e-e″). Phenotypes range from growth defects (f–f″) to catastrophic gonad defects (g–g″). Gonads outlined in white dashed lines. h) Representative image of gonad morphology in unmarked N2 animals hatched on *rab-1* RNAi (imaged 4 days post-maternal exposure) showing growth arrest and gonad morphology defects. Visible portion of the affected gonad in DIC is outlined in white dashed line. All scale bars = 20 μm. i) Graph showing percentage lethality and percentage body growth defects in larvae exposed to *rab-1* RNAi from the L1 stage for 72 h in both the marked strain shown in b)–d) (GFP::1NX-9; *mex-5p::H2B::mCherry::nos-2 3′UTR*) and the unmarked N2 strain. Error bars represent SE of the sample proportion (see Methods).

We probed the roles of Rab family genes in hermaphrodite gonad development (Fig. 1a), as the gonad is rapidly growing and contains proliferative germ cells (Roy *et al*. 2016) and large somatic cells (Byrd *et al*. 2014; Gordon 2020; Li *et al*. 2022), suggesting a significant dependence on intracellular trafficking. The gonad is also signal-active, with crucial regulatory interactions occurring between the soma and germline throughout life (Killian and Hubbard 2005; Kimble and Crittenden 2005; Gopal *et al*. 2021) and between the migrating somatic cells and the surrounding extracellular milieu during gonadogenesis (Meighan and Schwarzbauer 2007; Agarwal *et al*. 2022; Singh *et al*. 2024). We designed a conservative RNAi screening approach that circumvents early lethality to reveal postembryonic developmental requirements for a third of Rab genes—*rab5, rab-7*, *rab-10, rab-11.1, rab-11.2, glo-1, rab-6.2, rab-1, rab-19, rabr-2,* and C56E6.2*—*during gonad development in *C. elegans* hermaphrodites. Secondary screening of *rab-1* in tissue-specific RNAi strains reveals that *rab-1* is required cell-autonomously for proper gonad and vulval morphogenesis and noncell-autonomously for gamete formation. Surprisingly, the stem cell niche function of the distal tip cell (DTC) is not sensitive to the cell-autonomous *rab-1* knockdown that is sufficient to completely block gonad morphogenesis.

## Materials and methods

Sections of this text are adapted from prior Gordon lab publications (Li *et al*. 2022; Singh *et al*. 2024), as they describe our standard laboratory practices and equipment.

### RNAi

A single colony of *E. coli*  HT115(DE3) containing the L4440 plasmid with or without (L4440 RNAi control) a dsRNA trigger insert from the Ahringer (Kamath and Ahringer 2003) or Vidal (Rual *et al*. 2004) RNAi libraries, or our own clone in the case of *rab-14, glo-1* and Y71H2AM.12, was grown as an overnight culture containing ampicillin (100 μg/ml, VWR [Avantor], Chemical Abstracts Service (CAS) no. 69-52-3) at 37°C. Expression was induced with 1 mM IPTG (Apex BioResearch Products, CAS no. 367-93-1) for 1 h at 37°C, and 150–300 µl of induced RNAi culture was plated on NGM plates and allowed to grow on the benchtop at least overnight. Glycerol stocks were prepared from the preinduction overnight culture for storage at −80°C for future use, and a subsample was miniprepped and sent for sequencing to verify sequence of insert.

Worm populations were synchronized by bleaching according to a standard egg prep protocol (Stiernagle 2006), plated on NGM plates seeded with RNAi-expressing bacteria as arrested L1 larvae or in the maternal generation (as described below), and kept on RNAi until the time of imaging. See Supplementary Table 1 for full details about developmental staging and RNAi exposure time for all experiments.

### Initial Rab whole-body RNAi screening

Worms were maintained at 20°C. In the case of maternal RNAi exposure, 2–3 L4 hermaphrodites with somatic gonad membrane protein and germline nuclear markers (see Supplementary Table 3) were added to RNAi plates and allowed to feed on RNAi-expressing bacteria prior to egg laying. In the case of L1 RNAi exposure, synced L1s were plated on RNAi plates. Offspring were assessed for adult phenotypes 4 days later for maternal exposure and 72 h (3 days) later for L1 exposure, both at 20°C. *rab-1* maternal RNAi treatment resulted in high levels of embryonic lethality or larval arrest. In this case, RNAi was repeated by dropping egg-prepped L1 larvae directly onto *rab-1* RNAi plates and assessing adult phenotypes 72 h later. Each RNAi treatment was paired with an L4440 empty vector control treatment, none of which ever showed gonad growth defects. Therefore, an RNAi treatment that caused even a single incidence of gonad growth defect was counted as a “hit.”

### Tissue-specific and temporal RNAi of *rab-1*

The same *rab-1* RNAi clone used in the initial screen was rescreened in animals with tissue-specific RNAi activity. Germline-specific RNAi used a strain MAH23 carrying a mutation in *rrf-1(pk1417)*; it has some residual RNAi function in somatic cells (Kumsta and Hansen 2012). We validated germline sensitivity to RNAi in this strain with *glp-1* RNAi, which caused the expected high-penetrance defect of gonad growth (*n* = 36/44 gonads, see Supplementary Fig. 2).

A second tissue-specific strain has RNAi activity only in *lag-*2 promoter-expressing cells, namely the DTC, anchor cell (AC), and primary vulval precursors (see Supplementary Fig. 4). This strain, NK2115, carries an *rde-1(ne219)* loss of function that prevents RNAi activity globally, with RNAi function restored in the DTC and other cells (in an operon along with a coding sequence for membrane-tethered mNeonGreen) by a transgene *lag-2p::mNG::PLC^δPH^::F2A::rde-1* and a *rrf-3(pk1426)* mutation that enhances RNAi. RNAi treatment in this strain was conducted at 16°C due to the temperature-sensitive *rrf-3(pk1426)* mutation (Linden *et al*. 2017). A third tissue-specific strain, NK1316, has uterine-specific RNAi activity with *fos-1ap::rde-1* (Hagedorn *et al*. 2009; Matus *et al*. 2010) and functions by restoring RDE-1 protein activity in *rde-1(ne219)* mutant animals only to those cells of the somatic gonad that are under the control of the *fos-1a* promoter, expressed in uterine cells in the mid to late L2 stage. NK1316 also carries the *rrf-3(pk1426)* mutation that enhances RNAi, and fluorescent markers (see Supplementary Table 3).

### Scoring *rab-1* RNAi defects

**Fig. 2.**
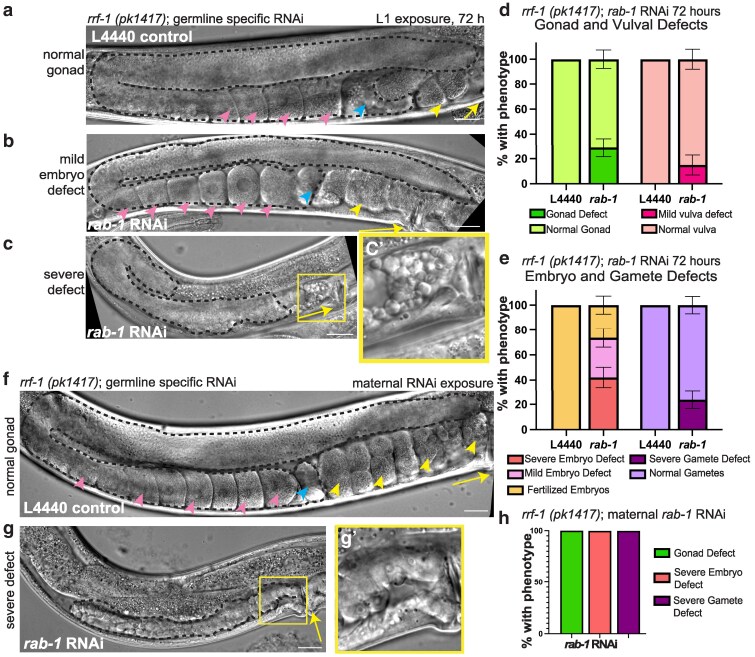
*C. elegans* with germline-specific *rab-1* RNAi knockdown develop gonads with embryo defects. Representative DIC images of MAH23 (*rrf-1(pk1417))* worms treated with L4440 control (a) or *rab-1* RNAi (b and c) for 72 h starting at the L1 stage. b) A portion of germline-specific *rab-1* RNAi-treated gonads have largely normal gonad morphology and gametes, but have disorganized, clumped, or misshapen embryos (classified as mild embryo defect, *n* = 12/38 gonads). c) Another portion of germline-specific *rab-1* RNAi-treated gonads have more severe gamete and embryo defects (severe embryo defect, *n* = 16/38 gonads). c′ shows the inset indicated by yellow box with abnormal gametes and no embryos. d) Quantification of gonad and vulva defects in germline-specific *rab-1* RNAi- and L4440 control-treated animals for 72 h from the L1 stage. *n* = 3/20 worms had a mild vulval defect (delay and pvl); *n* = 11/38 gonads had gonad defects (see Methods for scoring criteria). Error bars represent SE of the sample proportion. e). Quantification of embryo and gamete defects in germline-specific *rab-1* RNAi- and L4440 control-treated animals for 72 h from the L1 stage. *n* = 16/38 gonads had severe embryo defects, *n* = 12/38 had mild embryo defects, and *n* = 10/38 had normal/fertilized embryos. *n* = 9/38 gonads had severe gamete defects (see Methods for scoring criteria). Error bars represent SE of the sample proportion. Representative DIC images of MAH23 (*rrf-1(pk1417))* worms treated with L4440 control (f) or *rab-1* RNAi (g) imaged 4 days post-maternal exposure. g′) shows the inset indicated by yellow box with the absence of gametes and embryos. h) 100% of maternal *rab-1* germline-specific RNAi-treated gonads (*n* = 12/12 gonads) had severe gonad, gamete, and embryo defects. Yellow arrows mark the vulva, yellow arrowheads mark embryos, blue arrowheads mark sperm (spermatheca), and pink arrowheads mark oocytes. All scale bars = 20 μm. All gonads outlined in black dashed lines.

**Fig. 3.**
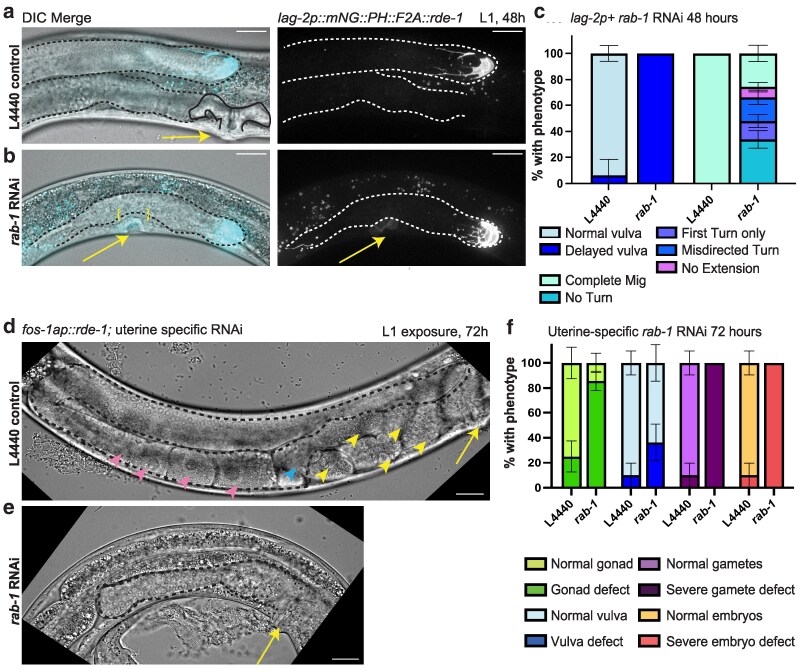
Somatic gonad-specific RNAi knockdown of *rab-1* causes numerous gonad defects. Representative images of *rde-1(ne219)* mutants rescued with a *lag2p::mNG::PLC^δPH^::F2A::rde-1* transgene restoring RNAi function and driving membrane-localized fluorescence protein mNeonGreen in cells that express the *lag-2* promoter on empty L4440 vector control (a) and *rab-1* RNAi (b) for 48 h after L1 arrest. Fluorescence merged with a single DIC *z*-slice (left), fluorescence alone (right, 8 μm maximum intensity projection displayed on a log scale to show DTC and dimmer VPCs, yellow arrows). Scale bars = 20 μm. Gonads outlined in dashed lines; control uterine lumen outlined in solid black line. Unbroken basement membrane in *rab-1* RNAi-treated (b) animal bracketed in yellow. c) Quantification of vulval (left) and DTC migration (right) defects in L4440 control and *rab-1* RNAi-treated animals following *lag-2p+-*specific RNAi for 48 h post-L1 exposure. (Left) Tissue-specific *rab-1* RNAi-treated worms (*n* = 17) have delayed vulva formation compared with controls (*n* = 16). For controls, *n* = 1/16 had not yet completed vulva formation, but *n* = 17/17 *rab-1* RNAi-treated worms had such a delay. (Right) Tissue-specific *rab-1* RNAi-treated worms (*n* = 50) have defective DTC migration compared with controls (*n* = 31). One gonad arm scored per worm. Growth of both gonad arms was typically affected, but the deeper DTC under the gut was difficult to score for orientation of turning. All control DTCs completed migration. For *rab-1* RNAi, *n* = 17/50 had no turn, *n* = 7/50 had just a first turn, *n* = 9/50 had a misdirected second turn, *n* = 4/50 had no extension after the second turn, and *n* = 13/50 completed migration. Error bars show SE of the sample proportion in c). Representative DIC images of uterine-specific (*fos-1ap::rde-1*) gonads treated with L4440 control (d) and *rab-1* RNAi (e) for 72 h post-L1 exposure. Yellow arrows mark vulva, yellow arrowheads mark embryos, blue arrowheads mark sperm (spermatheca), and pink arrowheads mark oocytes. Scale bars = 20 μm. Gonads outlined in black dashed lines. f) Quantification of gonad, vulval, gamete, and embryo defects in L4440 control and *rab-1* RNAi-treated animals represented in d) and e). In L4440 controls, *n* = 1/10 gonads had both severe embryo and gamete defect, *n* = 3/12 gonads had gonad defects, and *n* = 1/10 worms had a vulval defect. The variation in the total gonads scored is due to some gonads not having one of the scored features in frame. For *rab-1* RNAi-treated animals, *n* = 17/17 gonads had both severe gamete and embryo defects, *n* = 18/21 gonads had gonad defects, and *n* = 4/11 worms had vulval defects. Not every phenotype could be scored in every sample, causing variations in sample size within a treatment group. Error bars show SE of the sample proportion in f).

In histograms in Figs. 2 and 3 and Supplementary Fig. 2, RNAi-treated gonads that either failed to produce embryos or had severely deformed embryos were scored as having a severe embryo defect (see panel in Fig. 2c) and gonads that formed embryos that were clumped, disorganized, or misshapen were scored as having a mild embryo defect (see representative panel in Fig. 2b). Gamete phenotypes were scored as “severe gamete defect” (see representative panel in Fig. 2c and Supplementary Fig. 2b and d) if they either failed to produce gametes, produced severely deformed gametes, or had a severely reduced number of gametes. Gonads scored as having a “gonad defect” included samples that had severe gonad/germline growth defects (representative panel in Fig. 2c and Supplementary Fig. 2b and d) or those that have an overall gonad migration defect, or gonads that had both classes of defects (representative panel in Fig. 3e). Uterine defects were scored as absence of or deformed utse (uterine-seam) cell.

### Scoring DTC (gonad) migration and vulval defects

**Fig. 4.**
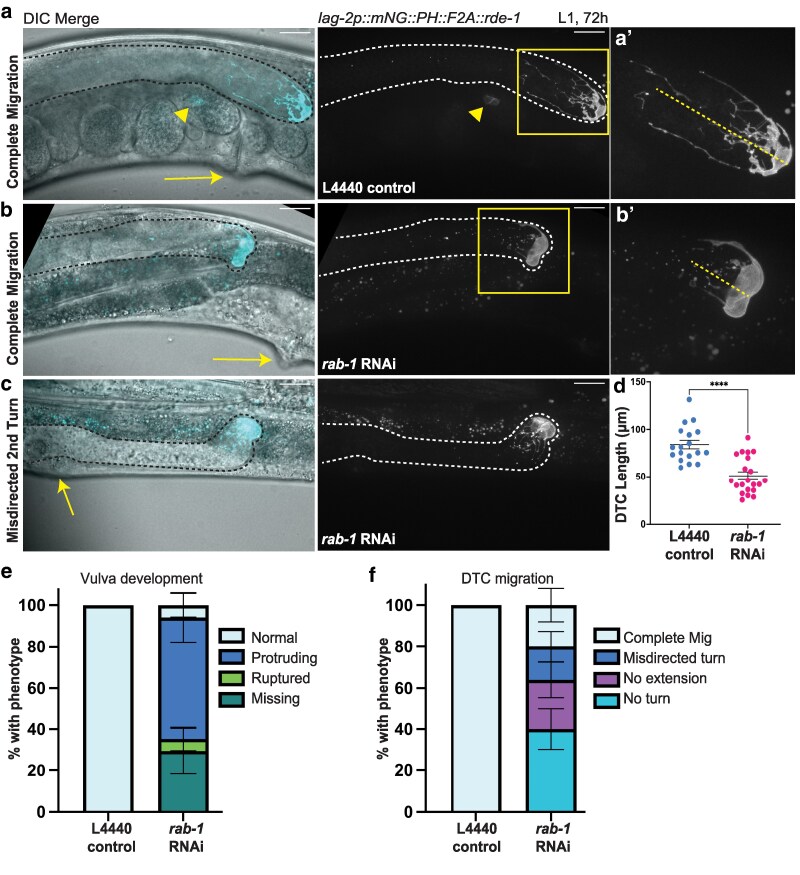
Reproductive system defects persist after prolonged *rab-1* RNAi knockdown in *lag-2* promoter-expressing somatic gonad cells. Representative images of reproductive age (72 h post-L1) animals with tissue-specific RNAi in cells expressing a *lag2p::mNG::PLC^δPH^::F2A::rde-1* transgene restoring RNAi function and driving membrane-localized fluorescence protein mNeonGreen on empty L4440 vector control (a) and *rab-1* RNAi (b, c) for 72 h after L1 exposure. Fluorescence merged with a single DIC *z*-slice (left), fluorescence alone (middle, maximum intensity projection through slices with mNeonGreen signal). a′) and b′) show insets indicated by yellow boxes. Scale bars = 20 μm. Gonads outlined in black or white dashed lines. Dashed yellow lines in a′) and b′) indicate length of DTC as measured for d). Yellow arrows (a–c) indicate vulva or site of expected vulval formation, showing normal vulva (a), protruding vulva (b), and vulvaless (c) phenotypes. Yellow arrowhead in a) indicates embryonic mNG expression. Quantification of *rab-1* tissue-specific RNAi-treated worm defects in DTC growth (d), vulva formation (e), and DTC migration (f) compared with L4440 control-treated animals following 72 h of RNAi exposure (from the L1 stage). Both gonad arms in the same worm were scored if both were visible. d) *rab-1* RNAi-treated worms (*n* = 21) have significantly shorter DTCs than controls (*n* = 18). Welch's *t*-test *t*(36.11) = 5.368, *P* < 0.0001, error bars show SEM. e) While *n* = 6/6 control samples had normal vulvas, only *n* = 1/17 *rab-1* RNAi samples had normal vulva, *n* = 5/17 had a missing vulva, *n* = 10/17 had a protruding vulva, and *n* = 1/17 ruptured through its protruding vulva on the slide. Error bars show SE of the sample proportion. f) While *n* = 18/18 control samples had complete DTC migration, only *n* = 5/25 *rab-1* RNAi samples completed migration, *n* = 10/25 failed to make any turns or elongate, *n* = 4/25 made the second turn in the wrong direction, and *n* = 6/25 made both turns and then failed to extend. Error bars show SE of the sample proportion.

To characterize the effects of *rab-1* RNAi knockdown in *lag-2* promoter-expressing cells on gonad (DTC) migration and vulval development in Figs. 3 and 4, DTC migration was scored by the following categories: CT: complete migration; NT: no turn; FT: first turn only; ST: second turn complete, but no extension; MD: misdirected second turn (Figs. 3 and 4).

Vulva formation was scored as wild type or delayed (large, round, *lag-2p::mNG*+VPCs still visible) at 48 h (Fig. 3) or wild type, protruding vulva (pvl), and vulvaless (vul) at 72 h (Fig. 4). DTC length was measured from tip to end of the longest process (Linden *et al*. 2017).

### Scoring DAPI-stained specimen

**Fig. 5.**
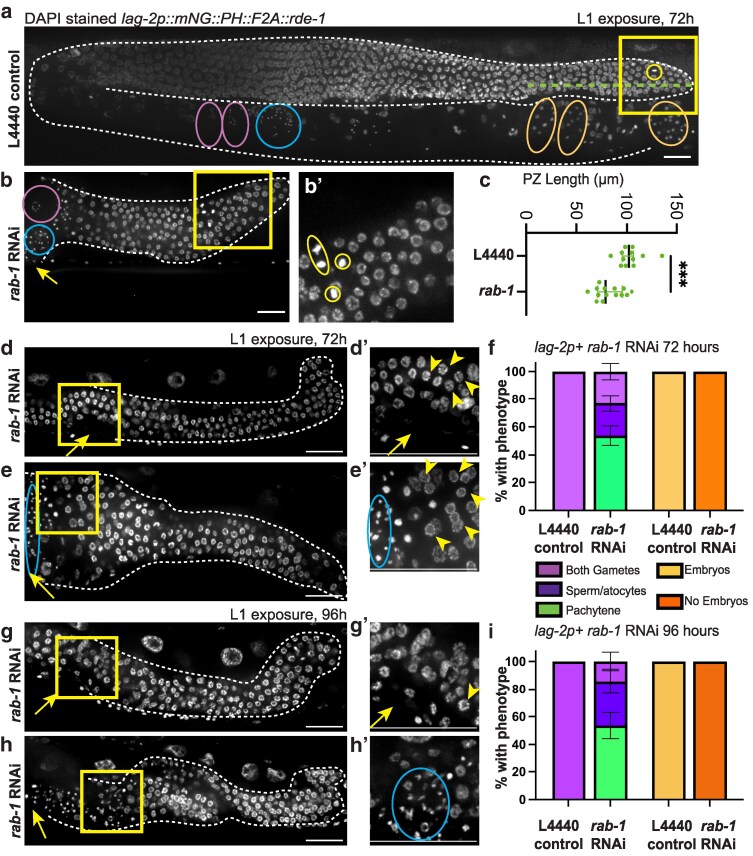
RNAi knockdown of *rab-1* in *lag-2* promoter-expressing somatic gonad cells causes germline proliferation and differentiation defects. Representative images of DAPI-stained reproductive age adult *rde-1(ne219)* mutants rescued with a *lag2p::mNG::PLC^δPH^::F2A::rde-1* transgene restoring RNAi function in cells that express the *lag-2* promoter on empty L4440 control (a) and the range of defects observed after tissue-specific *rab-1* RNAi for 72 h after L1 arrest (b, d, e). b′) shows inset indicated by yellow box (b). Dividing/mitotic germ cells are marked by yellow circle. Gametes, when present, are circled in pink (oocytes) and blue (spermatids). Embryos circled in orange. Dashed green line shows length of progenitor zone as measured for c). c) *lag-2p+ rab-1* RNAi-treated worms after 72 h following L1 RNAi exposure (*n* = 14) have shorter progenitor zones than control (*n* = 12), Welch's *t*-test, *t*(23.52) = 4.106, *P* = 0.0004; error bars show median with interquartile range. d) Pachytene arrest after 72 h on *rab-1* RNAi. d′) Same specimen in d) acquired with 1.6× optical zoom on region boxed in d). Yellow arrowheads indicate exemplar pachytene germ cells. Yellow arrow indicates site of expected vulva formation. e) Spermatogenesis after 72 h on *rab-1* RNAi. e′) Same specimen in e) acquired with 1.6× optical zoom of region boxed in e). Arrowheads indicate exemplar pachytene germ cells; spermatids circled in blue. Gonads outlined in dashed white lines. Yellow arrows indicate vulva or position of expected position of vulva formation, whenever in frame. Scale bars = 20 μm. f) Quantification of gametogenesis and embryo formation phenotypes in 72-h L4440 control- and *rab-1* RNAi-treated *lag-2p+* cell-specific RNAi animals represented in d) and e). f) Gamete (left) and embryo (right) defects observed for tissue-specific *rab-1* RNAi (*n* = 52 gonads) and L4440 control (*n* = 20 gonads). Error bars show SE of the sample proportion. 100% of L4440 control-treated gonads formed both gametes and embryos. For *rab-1* tissue-specific RNAi-treated animals, *n* = 28/52 gonads lacked gametes (germ cells in pachytene arrest), *n* = 12/52 gonads had both spermatids and oocytes, and *n* = 12/52 gonads had produced either spermatids or spermatocytes (developing sperm). *n* = 0/52 had formed embryos. g), h) Representative images of DAPI-stained *lag-2p+* cell-specific *rab-1* RNAi-treated worms after 96 h following L1 RNAi exposure. g) Pachytene arrest after 96 h on *rab-1* RNAi. g′) shows inset indicated by yellow box in g). Yellow arrowheads indicate exemplar pachytene germ cells. Yellow arrow indicates site of expected vulva formation. h) Spermatogenesis after 96 h on *rab-1* RNAi. h′) shows inset indicated by yellow box in h). Yellow arrow indicates site of expected vulva formation. Spermatids circled in blue. Gonads outlined in white dashed lines. i) Quantification of gametogenesis and embryo formation phenotypes in 96-h L4440 control- and *rab-1* RNAi-treated *lag-2p+* cell-specific RNAi animals represented in g) and h). Gamete (left) and embryo (right) defects observed for tissue-specific *rab-1* RNAi (*n* = 28 gonads) and L4440 control (*n* = 9 gonads). 100% of L4440 control-treated gonads had formed both gametes and embryos. For *rab-1* tissue-specific RNAi-treated animals, *n* = 15/28 gonads lacked gametes (germ cells in pachytene arrest), *n* = 4/28 gonads had both spermatids and oocytes, and another *n* = 9/28 gonads had produced either spermatids or spermatocytes (developing sperm). *n* = 0/28 had formed embryos. Error bars show SE of the sample proportion.

DAPI-stained adult worms (Fig. 5 and Supplementary Fig. 2g and h) were scored for presence of oocytes (large cells with chromosomes in diakinesis), spermatids (small pinpoints of DAPI), and embryos (multicellular structures in the proximal gonad). Progenitor zone was scored from the tip to the first row of germ cells with 2 crescent-shaped nuclei (Hubbard 2007). Some *rab-1* RNAi samples lacked an identifiable transition zone and were not scored for this measure. Mitotic figures were scored as a single bright metaphase plate or a pair of anaphase DAPI bodies; these were scored in the distal gonad and found to be absent in the proximal gonad.

### Confocal imaging

All images were acquired at room temperature on a Leica DMI8 with an xLIGHT V3 confocal spinning disk head (89 North) with a 63× Plan-Apochromat (1.4 NA) objective and an ORCAFusion GenIII sCMOS camera (Hamamatsu Photonics) controlled by microManager. RFPs were excited with a 555-nm laser; GFPs and mNGs were excited with a 488-nm laser; DAPI was excited with a 405 nm laser. *Z*-stacks through the gonad were acquired with a step-size of 1 µm unless otherwise noted. Worms were mounted on agar pads in M9 buffer with 0.01 M sodium azide (VWR [Avantor] CAS no. 26628-22-8l). Some samples were acquired with a 1.6× optical zoom, as indicated in the figure legends (Fig. 5d′ and e′).

### Image analysis

Images were processed in FIJI89 (version: 2.14.1/1.54f). Larger images tile several acquisitions of the same sample (Preibisch *et al*. 2009).

### Statistical analysis

Sample sizes vary slightly for measurements gathered from the same dataset if certain cell types were not clearly represented (for example, if the vulva is visible but the DTC is under the gut, or the image quality allows the DTC to be scored for position but not for length of processes). Sample sizes stated in figure legends or text reflect the number of samples analyzed for the specific feature being measured. Welch's 2-sample *t*-tests were used to compare *rab-1* RNAi to controls. SE of the sample proportion for the histograms in Figs. 1–5 and Supplementary Fig. 2 (after Levy-Strumpf *et al*. 2015) was calculated using the following equation:


$$SE\hat{\;p}=\sqrt{\frac{\hat{\;p}(1-\hat{\;p})}{n}}$$

where $\hat{\;p}$ is the percentage of specimen of the total observed *(n)* with the phenotype and error bars reflecting this calculation were added to plots using GraphPad Prism (Prism 10 for Mac OS; version 10.1.0 (264), 2023 October 18).

## Results and discussion

### Several Rab genes are required for the development of normal gonad morphology

We took an unbiased approach to characterizing roles for Rab GTPases in *C. elegans* gonad development (Fig. 1a) with a whole-body postembryonic RNAi screen in strains coexpressing a marker of germline nuclei and GFP::INX-9, a marker of the DTC and somatic gonadal sheath (see Supplementary Table 3) (Gordon *et al*. 2020; Li *et al*. 2022). This strain allows us not only to see overall gonad size and shape, but to resolve somatic cell structure and germ cell nuclear morphology after RNAi treatment (compare Fig. 1b–h). Because gonad development is a postembryonic process that culminates in reproductive adulthood, and there is documented lethality caused by loss of function for nearly half of *C. elegans* Rab genes (Table 1), we opted not to use a strain that is sensitized for whole-body RNAi. This makes our screen conservative and hopefully enriches for phenotypes caused by the loss of later-acting phases of Rab gene activity. We tested all 31 genes in the Rab family reported in a comprehensive phylogenetic analysis (Gallegos *et al*. 2012). We validated clones from the Ahringer RNAi library (Kamath and Ahringer 2003) for 25 Rab-encoding genes, the Vidal Unique library (Rual *et al*. 2004) for 3 genes (*rab-35*, *rab-19,* and C56E6.2), and generated our own clones for 3 genes (*glo-1, rab-14,* and Y71H2AM.12, Supplementary Table 2). The results from the screen are given in Table 1, along with a summary of the most severe loss-of-function phenotype for each gene reported on WormBase (Sternberg *et al*. 2024).

By analyzing knockdown phenotypes, we confirmed that loss of function of *rab-5* (Pushpa *et al*. 2021)*, rab-7* (Guo *et al*. 2010), and *rab-10* (Shi *et al*. 2010) causes gonad defects. We identified gonad defect phenotypes following knockdown of *rab-11.1, rab-11.2, glo-1, rab-6.2,* and *rab-1,* as well as 3 genes for which little functional data are currently available: *rab-19, rabr-2,*  C56E6.2 (Supplementary Fig. 1).

The Rab family genes that we found to cause gonad growth defects act in a range of processes. Only two, *rab-5* and *rab-7,* are previously known to act in the cells of the gonad. RAB-5 interacts with the PAR and exocyst complexes in the germline to control levels of GLP-1/Notch at the membrane that acts as the receptor of the DTC-expressed stemness cue LAG-2 (Pushpa *et al*. 2021). Evidence from *Drosophila* and mammalian cells indicates that RAB7 and RAB8 are required for proper localization of NOTCH1-GFP (Court *et al*. 2017). In *C. elegans,*  RAB-5 also acts with other Rab family members during engulfment of apoptotic cells; RAB-14, UNC-108/RAB-2, and RAB-7 follow RAB-5 recruitment and act sequentially in the formation of phagolysosomes in engulfing cells, including the gonadal sheath cells that engulf apoptotic germ cells (Guo *et al*. 2010). Knockdown of a RAB-7 guanine nucleotide exchange factor, *vps-45*, also causes defects in apoptotic germ cell engulfment (Kinchen *et al*. 2008).

Several of the Rab genes that cause gonad defects after RNAi have known roles in the *C. elegans* intestine: *rab-10, rab-11.1, rab-11.2*, and *glo-1*. The exocyst complex interacts with RAB-11 and RAB-10 in basolateral recycling and endosomal trafficking in the intestine (Shi *et al*. 2010; Chen *et al*. 2014; Li *et al*. 2024). The gene *glo-1* is required for the formation of lysosome-related organelles called gut granules (Hermann *et al*. 2005; Morris *et al*. 2018). Since the gonad is exquisitely sensitive to nutrient state (Templeman and Murphy 2018), noncell-autonomous gonad defects could be caused by breakdown in the gut-gonad trafficking and signaling axes. While some of these genes are also expressed in neurons (e.g. *rab-10,* Zou *et al*. 2015), RNAi is notoriously inefficient in neurons (Calixto *et al*. 2010), so we conclude that neuronal knockdown is unlikely to be the cause of the defects that we observe.

Previous studies report that *rab-8* (Cram *et al*. 2006) and *rab-6.2* (Shafaq-Zadah *et al*. 2015) RNAi cause defects in later phases of DTC migration. Our screen did not detect these migration phenotypes but did detect a gonad growth defect after *rab-6.2* RNAi. RNAi is inherently variable in its efficiency, so we do not consider this negative result to be in conflict with previous findings that these genes are required for DTC migration. Indeed, they suggest that our screen is conservative, as designed.

We also observed gonad growth defects after knockdown of 3 genes about which little is known: *rab-19, rabr-2,* and C56E6.2*. rab-19* is most closely related to human RAB19 and RAB43, and *rabr-2*/4R39.2 is homologous to human RAB44 (Gallegos *et al*. 2012). C56E6.2 does not have a clear human ortholog, but it has been reported to be transcribed in the somatic gonad precursors, Z1 and Z4 (Kroetz and Zarkower 2015). Finally, we found that normal gonad growth requires *rab-1*, the *C. elegans* paralog of human RAB1A and yeast YPT1, which is the founding member of the Rab family and key regulator of ER–Golgi transport.

### *rab-1* knockdown has profound effects on the germline that are cell autonomous and non-cell autonomous

The candidate we chose to pursue further is *rab-1*. We found that *rab-1* is important for gonad development, with *rab-1* RNAi causing severe defects in worms that survived to adulthood (Fig. 1a–i). Maternal *rab-1* RNAi caused a ~97% embryonic lethality as compared to controls (control progeny *n* = 275, *rab-1* RNAi progeny *n* = 8 larvae by day 3 after placing L4 mothers on plates and allowing them to lay). Most worms (marker strain shown in Fig. 1 b–d) and unmarked N2 animals (represented in Fig. 1h) treated from the L1 stage for 72 h with *rab-1* RNAi exhibited larval growth arrest or lethality by the L3 stage (Fig. 1i), which is to be expected given the early homozygous lethality of the balanced *rab-1(ok3750)* deletion allele (C. elegans Deletion Mutant Consortium, 2012). When we consider only worms that progressed through larval development after whole-body maternal *rab-1* RNAi, gonads were small, misshapen, had very few germ cells, and never laid embryos on the plate (Fig. 1c, d, and f–h). By screening in a genetic background expressing a marker of the somatic gonadal sheath and DTC (a tagged innexin protein, GFP::INX-9 (Gordon *et al*. 2020; Li *et al*. 2022), we can additionally observe that punctate membrane localization of INX-9 is impaired after *rab-1* RNAi (Fig. 1c″ and d″). A similar disruption of membrane protein signal was observed after *rab-1* RNAi in a genetic background expressing another tagged innexin, mKate::INX-8 (Gordon *et al*. 2020; Li *et al*. 2022) (Fig. 1f″ and g″). These tagged innexins and a DTC-expressed membrane-localized mNeonGreen::PLC^δPH^ reveal abnormal localization in the DTC and sheath after *rab-1* RNAi. While the DTC and somatic gonadal sheath are at a minimum present and in the correct relative positions—with the DTC at the tip and the gonadal sheath surrounding the germline—they have abnormal sizes, shapes, and membrane protein localization after *rab-1* RNAi. Wild-type N2 animals treated with maternal *rab-1* RNAi under the same conditions (Fig. 1h) also have major gonad morphology and growth defects (as well as body growth defects).

Rab1 regulates ER–Golgi trafficking generally (Plutner *et al*. 1991). However, in *Drosophila* clonal analysis (Charng *et al*. 2014) and S2 cells (Wang *et al*. 2010) Rab1 has been found to play more nuanced regulatory roles, including regulating Notch and integrin signaling. Such functions have been challenging to study in genetic loss-of-function mutants due to the critical role of Rab1 in basic cell function. Performing in vivo studies of this crucial gene in a developmental context can expand our understanding of how a highly conserved regulator of cellular processes can nonetheless play specific developmental roles.

To elucidate the tissue-specific functions of *rab-1*, we knocked down *rab-1* predominantly in the germline in a strain bearing an *rrf-1(pk1417)* mutation for a somatic RNA-directed RNA polymerase; this strain has RNAi efficacy in the germline and some RNAi activity in the soma, notably the gut (Kumsta and Hansen 2012). When animals are exposed to *rab-1* RNAi from the L1 stage, ∼29% had a gonad defect, primarily of gonad growth (Fig. 2a–d). Embryogenesis was notably impaired after germline-specific *rab-1* RNAi; over 70% of gonads had embryo defects, and over 20% of gonads had severe gamete defects (Fig. 2b, c, and e). Worms were also smaller than age-matched controls (Supplementary Fig. 3), potentially due to *rab-1* knockdown outside the germline. Germline defects may derive from aberrant trafficking of caveolin/CAV-1 after *rab-1* knockdown, a known role of *rab-1* in the germline (Sato *et al*. 2006). Caveolin trafficking requires both *rab-1* and *sar-1* (Sato *et al*. 2006), and we find that germline-specific *sar-1* RNAi phenocopies defects caused by germline-specific *rab-1* RNAi (Supplementary Fig. 2).

Maternal RNAi can sometimes be leveraged to get stronger knockdown of a gene of interest. Maternal germline-specific *rab-1* RNAi resulted in adult animals that formed very short gonads with abnormal germ cells and abnormal embryos (Fig. 2f–h and Supplementary Fig. 2). Maternal *sar-1* RNAi in this strain also caused fully penetrant gonad growth, gamete, and embryo defects in animals that reached adulthood (*n* = 10 gonads). These defects resemble maternal exposure to *glp-1*/Notch RNAi (Supplementary Fig. 2), in which the primordial germ cells Z2 and Z3 undergo a few divisions and then all germ cells exit the mitotic, stem-like state, as is known to happen after loss of function of Notch pathway genes in the germline (Fox and Schedl 2015).

Maternal exposure to RNAi in an RNAi strain with germline RNAi activity can lead to embryonic knockdown of targeted genes. Indeed, maternal *rab-1* RNAi treatment caused considerable embryonic lethality: out of 2 hermaphrodite parents put on *rab-1* RNAi, only 13 progeny escaped early larval arrest, of which 6 reached adulthood, while 2 parents on control RNAi produce hundreds of progeny. Maternal RNAi exposure in this strain therefore cannot distinguish between a specific requirement for *rab-1* in Z2/Z3 primordial germ cells or the larval germline vs a general requirement for *rab-1* in the developing embryo. We therefore focused on the most salient defects that manifest after L1 *rab-1* RNAi exposure in this strain, after which *rab-1* RNAi is more selective for the growing germline (Fig. 2a–e).

We determined that *rab-1* is required in the germline for gonad growth and the production of normal gametes and viable embryos, but most importantly we can conclude that loss of germline-specific *rab-1* function does not solely drive the dramatic gonad defects we see with whole-body *rab-1* knockdown (compare Fig. 2b and c with Fig. 1). We hypothesized that somatic gonad cells require *rab-1* function to properly regulate the germline, so we next investigated the role of *rab-1* in somatic gonad cells.

### *rab-1* RNAi knockdown in *lag-2p-*expressing somatic cells of the developing reproductive system affects gonad migration and growth, as well as uterus and vulva development

Since germline knockdown of *rab-1* does not recapitulate the gonad defects of whole-body *rab-1* RNAi knockdown, we hypothesized that *rab-1* may be required in the DTC for it to function as a germline stem cell niche and as the leader cell of gonad organogenesis. We performed *rab-1* knockdown in a strain considered to have DTC-specific RNAi activity (Linden *et al*. 2017; Agarwal *et al*. 2022; Singh et al. 2024). The strain carries an *rrf-3(pk1426)* RNAi-sensitizing mutation and an *rde-1(ne219)* loss-of-function mutation rescued by a *lag-2p::mNG::PLC^δPH^::F2A::rde-1* transgene restoring *rde-1-*dependent RNAi activity in sites of *lag-2* promoter expression, most notably the DTC, along with membrane fluorescence (Linden *et al*. 2017). Forty-eight hours after L1 exposure at 20°C on *rab-1* RNAi, DTC migration defects were seen in more than half of L4 larval animals (Fig. 3a, b, and c). These defects all involved the arrest of gonad elongation, often accompanied by failure to turn or misdirected turning, all defects that were absent in controls (Fig. 3a, b, and c). DTC migration requires signaling and adhesion, and proproliferative signaling from the DTC and gonadal sheath to the germ cells, proliferation of which provides the pushing force of migration (Agarwal *et al*. 2022). These DTC functions are mediated by cell membrane-bound receptors, and our results suggest they may be regulated by *rab-1*.

Surprisingly, these worms also lacked a well-differentiated uterine lumen or vulva (Fig. 3a–c). In wild-type worms, the vulva is patterned and connects to the uterus through a well-studied (Schindler and Sherwood 2013) series of inductive events and the invasion of the AC through the uterine and vulval basement membranes during the L3 larval stage (Katz *et al*. 1995; Sherwood and Sternberg 2003; Matus *et al*. 2010; Ihara *et al*. 2011; Morrissey *et al*. 2014). In L4 worms after 48 h on *rab-1* RNAi, we observe failure of AC invasion, with an intact basement membrane visible with DIC microscopy separating the gonad from the cells that should have formed the vulva (*n* = 15/17) and comparatively weak mNeonGreen expression in these vulval precursor cells (VPCs, Fig. 3b). VPCs are known to express *lag-2* (Zhang and Greenwald 2011); expression of the *lag-2p::mNG::PLC^δPH^::F2A::rde-1* transgene that restores RNAi function is an average of ∼50 × weaker in these VPCs than in the DTC at this stage, based on quantification of mNeonGreen expression (*n* = 16 L4 worms on *rab-1* RNAi with both vulval region and DTC captured). We see a delay in vulva formation in 17/17 of the *rab-1* RNAi-treated worms at this stage. We hypothesize that loss of *rab-1* function in the 1° VPCs prevents the completion of vulval development either through cell-autonomous defects in the 1° VPCs or the failure of these cells to signal to other cells, since VPCs engage in proinvasive signaling to the AC (Sherwood and Sternberg 2003) and production of Delta/Serrate/LAG-2 (DSL) signaling ligands to properly induce 2° VPC fate (Chen and Greenwald 2004).

There is no prior report of the effects of loss of *rab-1* on vulva development, but *rab-1* is required for proper development of the uterus, though its site of action in uterine development is not precisely known (Ghosh and Sternberg 2014). Uterine-specific *rab-1* RNAi, in strain NK1316 with *fos-1ap::rde-1* rescuing an *rde-1* loss of function and an *rrf-3(pk1426)* RNAi-sensitizing mutation (Hagedorn *et al*. 2009; Matus *et al*. 2010), causes profound defects of both uterus development and gonad growth and morphogenesis (Fig. 3d–f). The induction of both the 1° VPC fate and the uterine pi cell fate is regulated by signaling from the AC (Newman *et al*. 1996). The AC itself arises from 1 of 2 equipotent cells (Z1.ppp and Z4.aaa) that initially express *lag-2*/DSL ligand and *lin-12*/Notch. Via lateral inhibition between the 2, *lag-2* expression increases in 1 cell, which becomes the AC; the *lin-12-*expressing cell becomes the ventral uterine cell (Seydoux and Greenwald 1989). We confirmed that our *lag-2p::mNG::PLC^δPH^::F2A::rde-1* rescue transgene is transiently expressed in the AC, but not in the other uterine cells (Supplementary Fig. 4), meaning that in addition to early, continuous, and strong expression rescuing RNAi activity in the DTCs and weak expression in the VPCs, this strain also likely has transient RNAi activity in the AC. Later in wild-type development, the AC fuses with a subset of descendent cells of the uterine pi lineage to become the uterine-seam cell (utse) (Newman *et al*. 1996), so it is possible that the AC could carry RNAi activity via RDE-1 protein into the utse upon fusion. Alternatively, by disrupting induction of the uterine pi cell fate by the AC, RNAi activity in the AC could prevent proper differentiation of uterine cell types.

Just as *rab-1* RNAi in the vulva could affect proinvasive signaling to the AC, knockdown of *rab-1* in the AC could also cause defects in vulval development. For example, vulval defects are observed if the AC fails to induce the primary vulval fate, fails to pattern the descendants of the 1° VPCs (Wang and Sternberg 2000), or otherwise fails to invade and connect the uterus and vulva (Sherwood and Sternberg 2003). In a prior RNAi screen for genes acting in the AC to regulate cell invasion that used a uterus-specific RNAi strain, *rab-1* was not tested (Matus *et al*. 2010). When we expose the uterine-specific RNAi strain to *rab-1* RNAi from the L1 stage, we find high penetrance of uterine defects (*n* = 9/10), no vulvaless worms, and few (*n* = 4/11) with more minor vulva defects (Fig. 3d–f). We conclude that severe defects in vulval morphogenesis are probably caused by *rab-1* RNAi in the VPCs themselves, which express the *lag-2p::mNG::PLC^δPH^::F2A::rde-1* RNAi rescue gene (Supplementary Fig. 4).

### Prolonged tissue-specific *rab-1* RNAi knockdown in somatic gonad cells impedes vulva formation, DTC niche maturation, and germ cell proliferation

Worms with RNAi activity in *lag-2* promoter-expressing somatic cells treated with *rab-1* RNAi were also smaller than those receiving control RNAi empty vector treatment (Supplementary Fig. 3). We next asked whether development was simply delayed, as whole-body *rab-1* RNAi is documented to cause developmental delay (Ghosh and Sternberg 2014), or whether gonad defects would remain with prolonged *rab-1* RNAi treatment. We allowed animals to continue to develop on *rab-1* RNAi until the age-matched controls had reached reproductive adulthood (72 h after being released from L1 arrest at 20°C, Supplementary Table 1).

The penetrance of vulval morphology defects remained high after prolonged *lag-2* promoter-expressing cell-specific *rab-1* knockdown, though they progressed from the “delay” phenotypes in which VPCs expressing *lag-2p::mNG* could be easily identified in adults to phenotypes like protruding vulva (pvl) or missing vulva/vulvaless (vul) (Fig. 4a–c and e).

The penetrance of the DTC migration defects also remained high after 72 h (Fig. 4a–c and f), demonstrating that gonad migration and elongation do not recover over developmental time after knockdown of *rab-1* in *lag-2* promoter-expressing cells. These results are strong evidence that *rab-1* knockdown in the DTC is interfering with gonad migration, not simply global progression of development. Proper migration requires germ cell proliferation-driven gonad growth (Agarwal *et al*. 2022) and turning, which is regulated by several signaling pathways (Levy-Strumpf *et al*. 2015; Singh *et al*. 2024) and interactions with the basement membrane (Agarwal *et al*. 2022).

Upon reaching reproductive age, animals with RNAi activity in *lag-2-*expressing somatic cells also developed germline defects. Worms had smaller DTCs (Fig. 4d) and smaller germline proliferative zones (Fig. 5a–c). We also observed fewer actively dividing cells after *rab-1* RNAi, with an average of 1.67 divisions in *rab-1*-RNAi-treated worms and 3.58 dividing cells in the controls (Welch's 2-sample *t*-test, *t* = 2.6752, df = 23.992, *P* = 0.01324, 95% CI = 0.438–3.395). However, more than half of the RNAi-treated worms had mitotic figures (Fig. 5b–b′), and in no case did we observe evidence of germ cell differentiation at the distal end of the gonad, which is the expected phenotype if DTC-expressed LAG-2/DSL protein is not able to signal through GLP-1/Notch receptors on the distal germ cells (Cinquin *et al*. 2010; Fox and Schedl 2015). Continued maintenance of germ cells with the mitotic fate after prolonged *rab-1* RNAi in *lag-2* promoter-expressing cells suggests that localization of the stemness cue LAG-2 to the DTC membrane is not as sensitive to *rab-1* knockdown as factors regulating DTC migration behavior.

### A somatic signal that promotes pachytene exit and gamete differentiation depends on *rab-1* activity in a *lag-2* promoter-expressing cell

Scoring the length of the proliferative zone in DAPI-stained samples revealed 4/18 samples lacking a discernible transition zone, in which germ cells in early meiotic prophase have a distinctive crescent-shaped nuclear morphology (Hubbard 2007). The majority of animals also fail to make gametes—both sperm and eggs—normally (Fig. 5d, e, and f). When a germline is shorter than normal, meiotic entry delay can result because much of the germline remains within niche signaling range of the DTC (Kimble and White 1981). There is also a “latent niche” DSL ligand signal from the proximal gonad that can support germline mitotic fate if undifferentiated germ cells come in contact with the proximal gonad (McGovern *et al*. 2009). However, we did not observe proximal mitotic figures in these very small gonads (as in pro mutants, McGovern *et al*. 2009), and most samples did show evidence of meiotic entry with a well-demarcated transition zone, suggesting that germ cells are escaping the niche signal (Fig. 5c, *n* = 14/18).

Instead, we propose that the phenotypes we observed after prolonged *rab-1* RNAi knockdown in *lag-2* promoter-expressing cells represent failure to progress through meiotic pachytene (Church *et al*. 1995; McCarter *et al*. 1997; Lee *et al*. 2007). After 72 h on *rab-1* RNAi in cells expressing the *lag-2* promoter, the majority of gonads (*n* = 28/52) exhibited nuclear signal consistent with meiotic pachytene in the proximal-most germ cells (Fig. 5d–d′ and f). Of the remaining samples, 21/52 gonads had formed spermatids (Fig. 5e–e′ and f) and 3/52 gonads had morphology consistent with late stages of male gamete meiosis (Shakes *et al*. 2009), but lacked spermatids (Fig. 5f). Of the 21 gonads with spermatids, 12 also had DAPI signal consistent with at least 1 oocyte (Fig. 5f). Fertilized embryos were never observed (Fig. 5f, *n* = 0/52 gonads), while all control samples had fully differentiated gametes and fertilized embryos in each gonad arm (sperm and oocytes; *n* = 20/20 gonads).

At 96 h post-L1 arrest (24 h later, see Supplementary Table 1), the same proportion of tissue-specific *rab-1* RNAi samples were arrested in meiotic pachytene (Fig. 5i). These samples also lack evidence of vulva formation (while samples with spermatids often have protruding vulvas). It is possible that this difference reflects a developmental arrest of some animals in a pregametogenic state, though their body growth clearly surpasses that of L3 larvae. We favor the interpretation that this difference reflects variability in *rab-1* RNAi knockdown efficiency and therefore that the strongest *rab-1* knockdown in *lag-2* promoter*-*expressing cells causes a block of both gametogenesis and vulvagenesis. Supporting this conclusion, *sar-1* RNAi in *lag-2* promoter-expressing cells cause a near complete pachytene arrest (Supplementary Fig. 2g and h, *n* = 35/36 gonads, with 18/18 worms lacking a vulva).

A defect of pachytene exit affecting both spermatogenesis and oogenesis with similar penetrance was observed in a prior study after laser ablation of both sheath–spermathecal (SS) progenitor cells in a gonad arm (57% of gonads had sperm, only 4% of arms made a single oocyte, McCarter *et al*. 1997). The genetic regulation of meiotic cell cycle progression is complex, especially for the oogenic germline (Arur 2017), in which MPK-1 (the *C. elegans* ortholog of ERK, the primary MAPK required for germline differentiation) signaling plays a crucial role (Lee *et al*. 2007; Das and Arur 2022). MPK-1/MAPK signaling regulates the germline both cell-autonomously (Lee *et al*. 2007) and noncell-autonomously (Robinson-Thiewes *et al*. 2021). Nutritional inputs also regulate MPK-1/MAPK-mediated progress through meiosis in the oogenic germline through the insulin-like receptor encoded by *daf-2*, with neuronal insulin-like peptides proposed as the signals that activate germline DAF-2 (Lopez *et al*. 2013). The source and identity of the somatic gonad signal that promotes pachytene exit have not yet been identified.

We propose that by knocking down *rab-1*—and thereby a key step in the production of secreted and membrane-bound proteins—in *lag-2* promoter-expressing cells of the reproductive system, we have identified a subset of cells (DTC, SS cells [see below], AC, vulval cells) within which may be the source of a somatic gonad pachytene exit signal, or at least a cell required for the proper development of the source of that signal (e.g. the AC is required for development of the uterus in the proximal gonad).

Considering the proximal gonad, after *rab-1* RNAi in our “uterine-specific” RNAi strain, we also see a penetrant loss of differentiated germ cells and embryos (Fig. 3d–f). The *fos-1* promoter is strongly expressed in the cells of the spermatheca in addition to the uterine cells (www.wormseq.org). Ablation of dorsal uterine cells did not cause pachytene exit defects (McCarter *et al*. 1997). We therefore favor the possibility that early *rab-1* RNAi in the SS lineage is the likely point of convergence between the pachytene arrest phenotype we observe and that observed after cell ablations in the somatic gonad lineage.

Some *lag-2* promoter-driven transgenes that activate in Z1 and Z4, the somatic gonad progenitor cells, show residual expression in other somatic gonad cell types later in development like the somatic gonad sheath cells (Blelloch *et al*. 1999; Killian and Hubbard 2005). We saw expression of the *lag-2p::mNG::F2A::rde-1* rescue transgene in Z1 and Z4 and then in the SS progenitor cells for a brief time in the L2 larval stage (Supplementary Fig. 4). The SS lineage is the only point of convergence between *lag-2* promoter-expressing cells and cells targeted by the SS ablation experiment that blocked pachytene progression (McCarter *et al*. 1997), though the *lag-2* promoter is active in the SS cells for a very brief time (Supplementary Fig. 4). Further work on the sensitive developmental window in which *rab-1* RNAi in *lag-2* promoter-expressing cells inhibits pachytene progression may narrow down the source of a somatic signal necessary for gamete differentiation.

### Conclusion

We found that 11 of the 31 Rab GTPase-encoding genes in *C. elegans* play a role in gonad development; these numbers are conservative, as RNAi can only implicate a gene in a developmental process, not rule out its participation. In particular, *rab-1* regulates development of the somatic gonad and germline in both cell-autonomous and non-cell-autonomous ways. Neither germline-specific *rab-1* RNAi nor *rab-1* RNAi in *lag-2* promoter-positive cells fully recapitulated the catastrophic gonad and germline defects we observed after whole-body *rab-1* RNAi treatment (Fig. 1), so we conclude that systemic *rab-1* is essential for germline and gonad growth. In the future, intestinal *rab-1* should be examined for a role in gonad development, as several known intestine-expressed Rab genes (*rab-10, rab-11.1, rab-11.2*, and *glo-1*) also caused gonad defects when knocked down with whole-body RNAi (Table 1). Tissue-specific RNAi implicates cell-autonomous *rab-1* in the formation of normal embryos, vulva morphogenesis, the proper development of uterine cells, and DTC migration. We find new evidence of RNAi activity during development in cells that transiently or weakly express the promoter rescuing RNAi function in a purportedly cell type-specific RNAi strain, which is important for the use of this strain in the future. The germline requires noncell-autonomous *rab-1* expression for normal proliferation and for pachytene exit. Surprisingly, the least-sensitive feature of the reproductive system to *rab-1* RNAi knockdown is the stem cell niche function of the DTC, which survives even strong knockdown of *rab-1* in the DTC. This study motivates future investigations into the role of *rab-1*-independent signaling from the stem cell niche to the germline, as well as *rab-1*-mediated signaling to promote pachytene exit.

## Supplementary Material

jkaf085_Supplementary_Data

## Data Availability

The authors affirm that all data necessary for confirming the conclusions of the article are present within the article, figures, and tables. Strains and plasmids are available upon request. Supplemental material available at G3 online.
