# Supplementary material for: An RNAi screen of Rab GTPase genes in Caenorhabditis elegans reveals that morphogenesis has a higher demand than stem cell niche maintenance for rab-1 in the somatic cells of the reproductive system
Source: G3 (Bethesda). 2025 Apr 16;15(6):jkaf085. doi: 10.1093/g3journal/jkaf085 (PMC12135009; doi:10.1093/g3journal/jkaf085)
Supplement: jkaf085_Supplementary_Data [file jkaf085_supplementary_data.pdf]

Figure S1. Whole-body RNAi of three uncharacterized Rab GTPase family genes causes gonad defects.

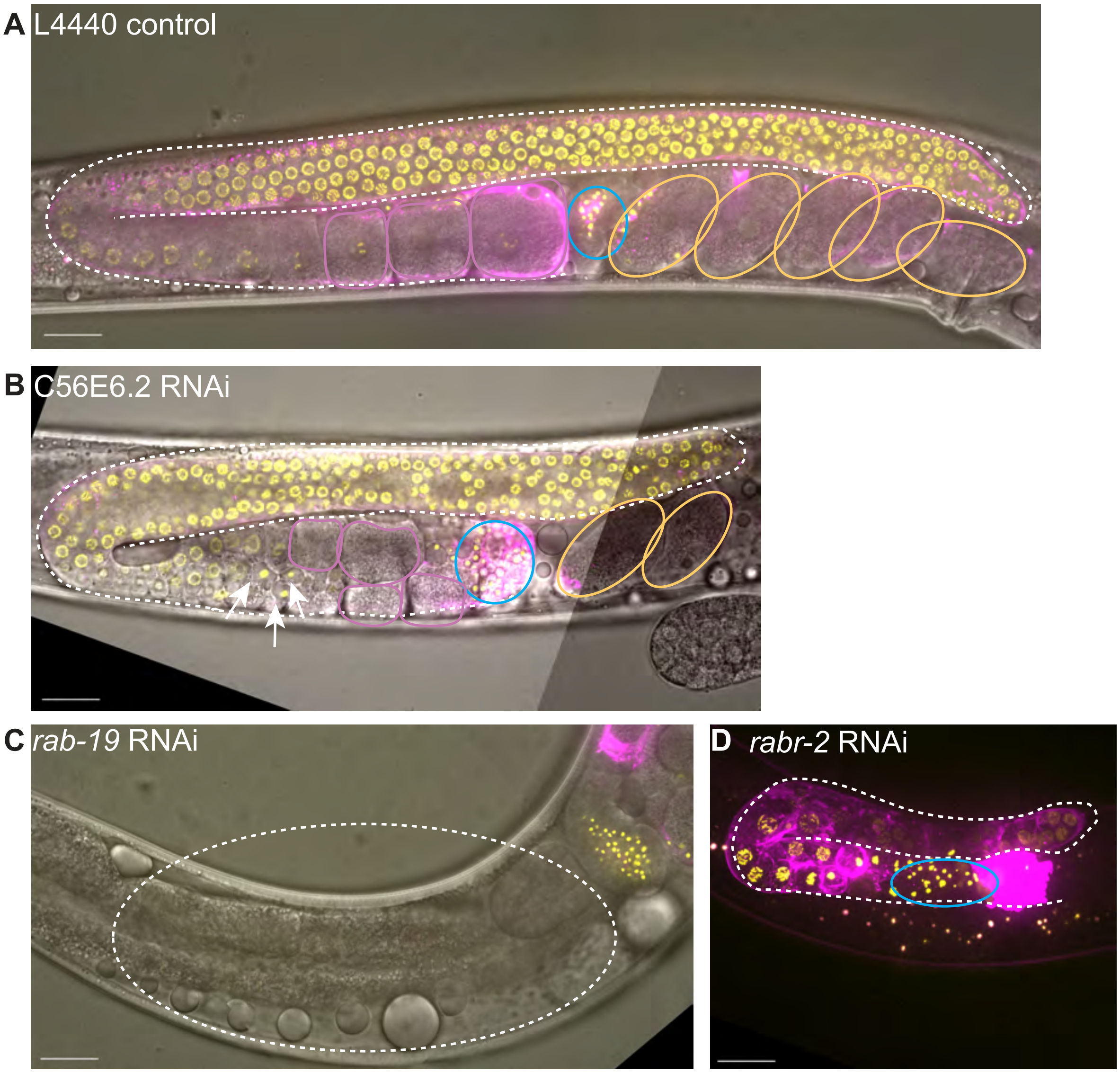

Figure S1. Whole-body RNAi of three uncharacterized Rab GTPase family genes causes gonad defects. Representative images of germ cells (yellow; *mex-5p::H2B::mCherry::nos-2 3'UTR*) and somatic gonad DTC and sheath cells (magenta; GFP::INX-9) in animals exposed to (A) RNAi control, (B) C56E6.2 RNAi, (C) *rab-19* RNAi, and (D) *rabr-2* RNAi from the L1 stage. Oocytes outlined in pink, sperm circled in blue, and embryos outlined in orange. White arrows in (B) indicate abnormal gamete formation in which cells resembling primary spermatocytes are still observed in an otherwise oogenic germline. White dashed outlines indicate focal gonad arms, with the white dashed circle in (C) indicating missing posterior gonad arm. Scale bars = 20 µm.

Figure S2. Tissue-specific *sar-1* and *glp-1* RNAi phenocopies *rab-1* RNAi.

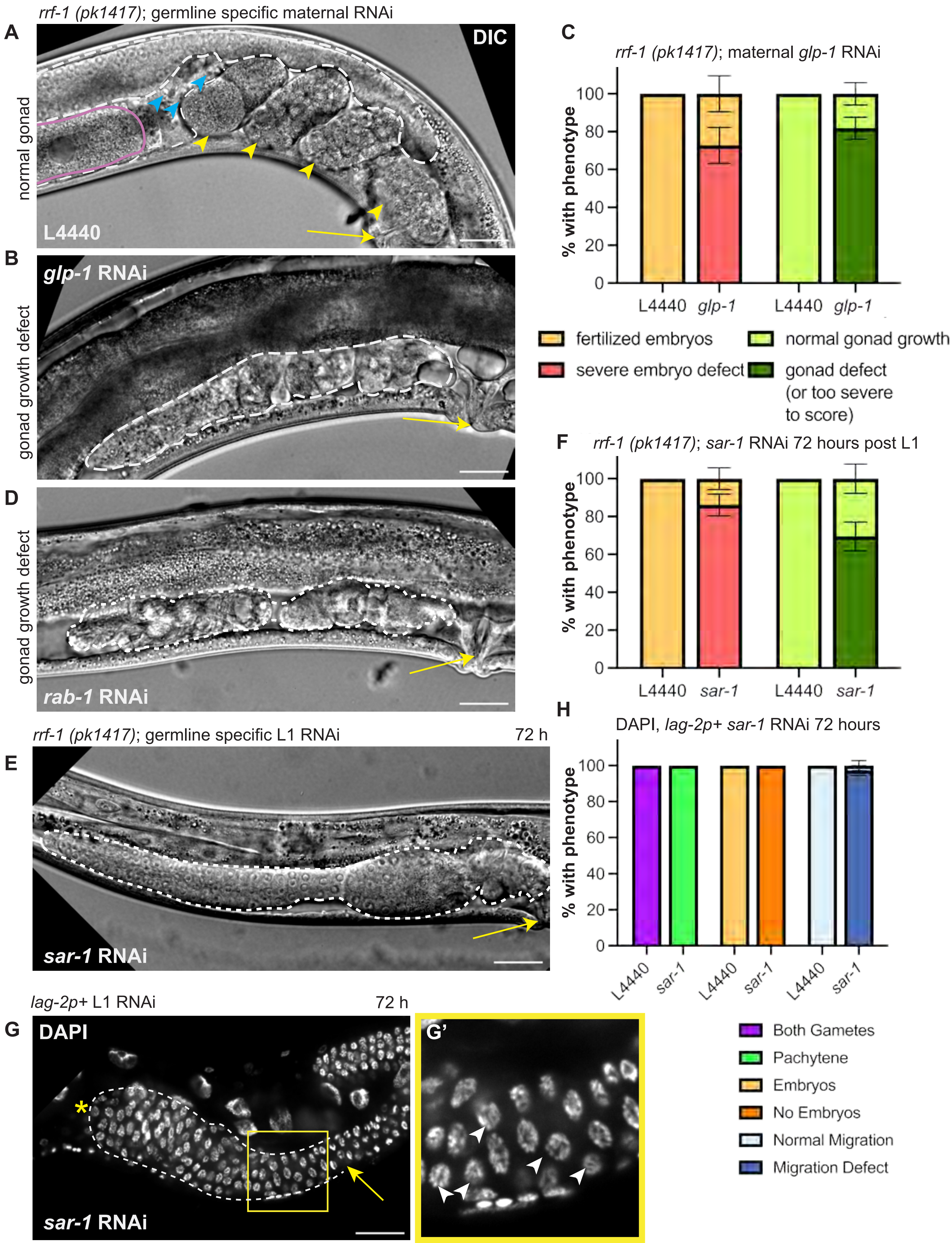

Figure S2. Tissue-specific *sar-1* and *glp*-1 RNAi phenocopies *rab-1* RNAi. (A-B, D) Representative DIC images of MAH23 *rrf-1 (pk1417)* (germline-specific RNAi) animals treated maternally with L4440 control (A), *glp-1* (B) and *rab-1* (D) RNAi (imaged 4 days post maternal exposure). Both *glp-1* (B) and *rab-1* (D) maternally treated RNAi animals had severe gonad growth, gamete and embryo formation defects. (C) Quantification of embryo (n=32/44 gonads) and gonad defects (n=36/44 gonads) in *glp-1* maternally treated MAH23 *rrf-1(pk1417)* (germline-specific RNAi) animals represented in B. (*rab-1* maternal exposure phenotypes quantified in Fig. 2h). (E) Representative DIC image of MAH23 *rrf-1(pk1417)* (germline-specific RNAi) animals treated with *sar-1* RNAi (imaged 72 hours post L1 RNAi exposure). (F) Quantification of embryo and gonad defects (legend above shared with C) in L4440 (n=18 gonads) and *sar-1* RNAi treated (n=36 gonads) *rrf-1 (pk1417)* (germline-specific RNAi) animals at 72 hours post L1 RNAi exposure. n = 31/36 *sar-1* RNAi treated gonads had severe embryo defects, and n = 25/36 gonads had gonad defects (which includes n=3/36 severely deformed gonads). (G) Representative image of DAPI-stained *rde-1(ne219)* mutants rescued with a *lag2p::mNG::PLC*$^{\delta PH}$*::F2A::rde-1* transgene restoring RNAi function in cells that express the *lag-2* promoter on *sar-1* RNAi (exposure for 72 hours post L1 arrest; L4440 control sample not shown). Yellow asterisk in G marks the gonad tip. G' shows inset indicated by yellow box. White arrowheads in G' show exemplar pachytene nuclei in the proximal gonad. (H) Quantification of gamete, embryo and gonad migration phenotypes in DAPI stained *lag-2p*+ cell-specific *sar-1* RNAi treated animals (represented in G). n=8/8 young-adult L4440 treated gonads had both gametes, embryos and had normal gonad migration whereas stage-matched *sar-1* treated animals had n=0/35 gonads with spermatids or developing sperm, n=0/35 gonads with oocytes, and n=35/35 gonads with germ cells in pachytene arrest; n=0/35 gonads had embryos, and n=35/36 showed abnormal gonad migration. 1 of 36 *sar-1* RNAi treated gonad was too severe to score for some features (gametes and embryos) but was scored for gonad migration. Error bars in C, F, H represent the standard error of the sample proportion. Scale bars for all panels = 20 µm. Gonads outlined in white dashed lines. Yellow arrows mark the vulva (A-E) or expected region of vulval formation (G). Yellow arrowheads mark embryos, blue arrowheads mark sperm (spermatheca), pink outline in A outlines visible oocytes in frame.

Figure S3. RNAi knockdown of *rab-1* in the germline and *lag-2* promoter-expressing cells results in small body size at the end of larval development

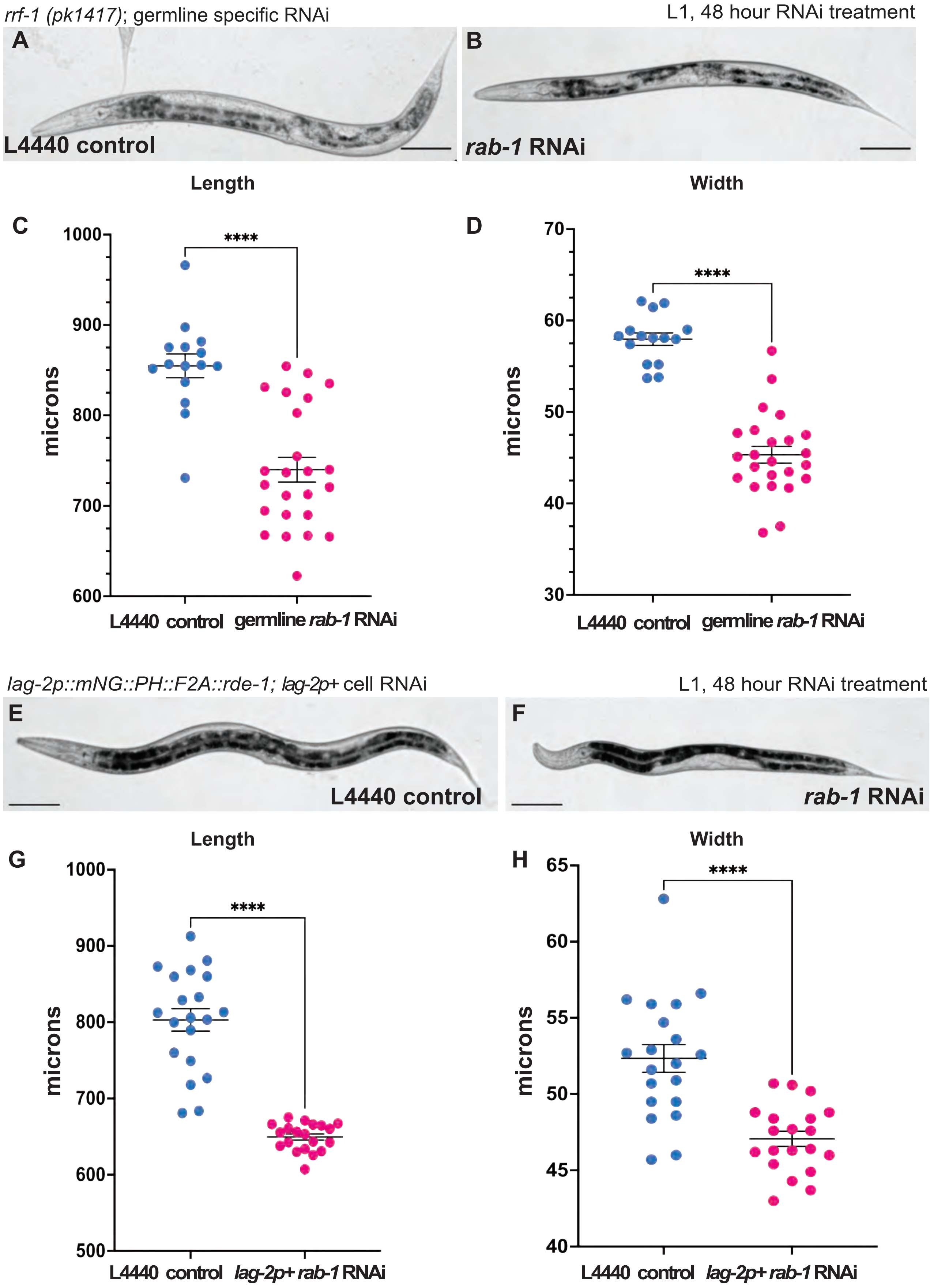

Figure S3. RNAi knockdown of *rab-1* in the germline and *lag-2* promoter-expressing cells results in small body size at the end of larval development. (A-B) Representative 20x brightfield images of young adult germline-specific MAH23 (*rrf-1(pk1417))* L4440 control (A) and *rab-1* RNAi treated (B) worms for 48 hours from L1 RNAi exposure; (scale bars = 100 μm). (C-D) MAH23 (*rrf-1(pk1417))* worms treated with *rab-1* RNAi (n=24) have decreased body length (C) and width (D) compared to L4440 treated controls (n=15). Statistical significance was calculated by unpaired, two-tail Student's t-tests, error bars represent ±SEM, and **** denotes *p*-value < 0.0001. (E-F) Representative 20x brightfield images of young adult *lag-2* promoter expressing cell-specific RNAi animals treated with L4440 control (E) and *rab-1* RNAi (F) for 48 hours from L1 RNAi exposure; (scale bars = 100 μm). (G-H) *lag-2p*+ specific RNAi worms treated with *rab-1* RNAi (n=20) have decreased body length (G) and width (H) compared to controls (n=20). Mean ± S.E.M graphed in G and H. Statistical significance was calculated by unpaired, two-tail Welch's t-tests. Length $t(21.82)=10.03$, $p<0.0001$, width $t(29.39)=5.120$, $p<0.0001$.

Figure S4. Locations of *lag-2p::mNG::F2A::rde-1* rescue transgene expression

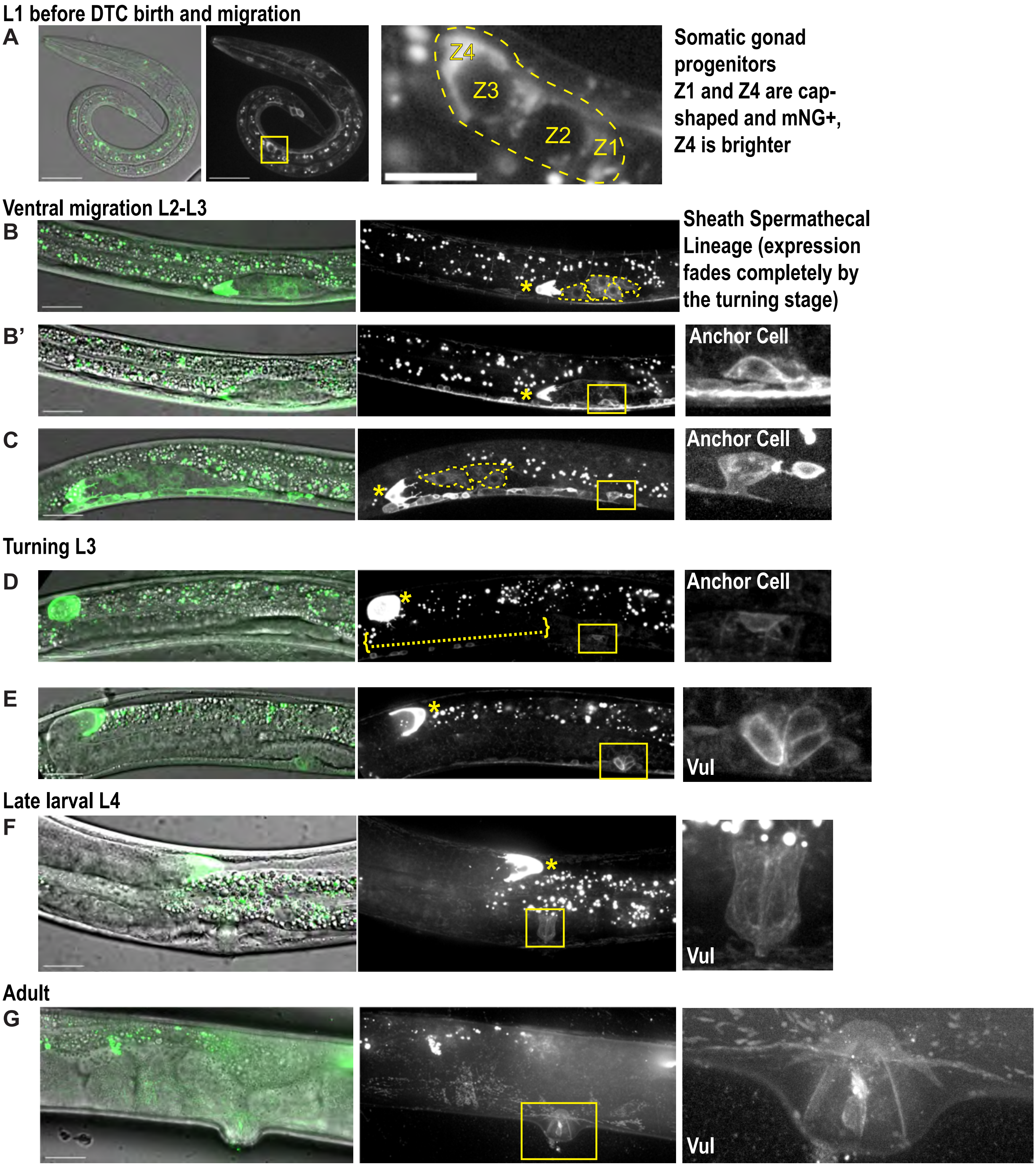

Figure S4. Locations of *lag2p::mNG::F2A::rde-1* rescue transgene expression during development. Representative images of the strain bearing the *lag2p::mNG::PLC*$^{\delta PH}$*::F2A::rde-1* allele (with mutations in *rrf-3(pk1426)* and *rde-1(ne219)*) in the L1 (A), L2 (B-C), turning/late L3 (D-E), L4 (F), and Adult (G) life stages in which RNAi is presumably active in mNG+ cells (with the exception of neurons, which are not sensitive to RNAi (Calixto et al., 2010), seen along the ventral body wall in some images and in superficial circumferential projections in B). Single slices or projections through 0.5-2.5 microns from confocal z-stacks, DIC images merged with green fluorescence (left), and green fluorescence alone (grayscale, center and insets). Imaged with 0.5 micron step size, except (A), which was imaged with 0.2 micron step size. Greyscale images shown with fluorescence logarithmically scaled to enhance visibility of dim signal. Boxes show region of inset. Asterisks mark distal tip. (A) Somatic gonad progenitor cells Z1 and Z4 express mNG in L1 arrested larvae, with substantially more expression in Z4 than Z1; primordial germ cells Z2 and Z3 do not express the rescue transgene. (B) In the L2, cells of the sheath-spermathecal (SS) lineage (outlined in yellow dashed lines) are mNG+ early, possibly as residual expression from the somatic gonad precursor cells; these cells are no longer visible by the turning stage in L3 (brackets in D). (B') Projection through deeper Z-slices of the same sample in B. The anchor cell (AC) is mNG+ from the late L2 when it is first born. (C) SS cells are still faintly mNG+ at the time of AC invasion (inset). (D) By the time of DTC turning in the L3, no SS expression is observed, and signal at the future site of vulva formation (inset) is very dim. (E) As the turn is completed, the vulval precursor cells (inset) are mNG+. (F) The vulva remains faintly mNG+ in L4 larvae and (G) adults, note mild pvl phenotype. Scale bars = 20 μm.

Table S1. Stages and RNAi-exposure conditions for each experimental figure panel

Item	Panel	Experiment	site of action	RNAi exposure	Imaging/quantification stage
Table 1		somatic gonad; germ-cell histone marker (GFP::INX-9; *mex-5p::*H2B::mCherry) on RNAi for each Rab family protein	whole-body	As noted in Table	4 days post exposure; (stage-matched controls reached young adulthood)
Fig. 1	b-d	somatic gonad; germ-cell histone marker (GFP::INX-9; *mex-5p::*H2B::mCherry) on *rab-1* RNAi	whole-body	maternal	4 days post exposure; (controls reached young adulthood)
Fig. 1	e-g	somatic gonad marker (mKate::INX-8; *lag-2p::*mNG) on *rab-1* RNAi	whole-body	maternal	4 days post exposure; (controls reached young adulthood)
Fig. 1	h	N2 (wildtype) strain on *rab-1* RNAi	whole-body	maternal	4 days post exposure; (controls reached young adulthood)
Fig. 1	i	*rab-1* RNAi lethality and growth phenotype quantification in marker strain vs. N2	whole-body	L1	72h post L1; (stage-matched controls are young adults)
Fig. 2	a-e	germline-specific *(rrf-1(pk1417))* on *rab-1* RNAi	tissue-spec.	L1	72h post L1; (controls are young adults)
Fig. 2	f-g	germline-specific *(rrf-1(pk1417))* on *rab-1* RNAi	tissue-spec.	maternal	4 days post exposure; (controls reached young adulthood)
Fig. S2	a-c	germline-specific *(rrf-1(pk1417))* on *glp-1* RNAi	tissue-spec.	maternal	4 days post exposure; (controls reached young adulthood)
Fig. S2	d	germline-specific *(rrf-1(pk1417))* on *rab-1* RNAi	tissue-spec.	maternal	4 days post exposure; (controls reached young adulthood)
Fig. S2	e-f	germline-specific *(rrf-1(pk1417))* on *sar-1* RNAi	tissue-spec.	L1	72h post L1; (controls are young adults)
Fig. S2	g-h	DAPI stain of *lag-2* promoter specific RNAi (*lag-2p::mNG::PH::F2A::rde-1)* on *sar-1* RNAi	tissue-spec.	L1	72h post L1; (controls are young adults)
Fig. S3	a-d	body size of *rab-1* RNAi exposured germline-specific *(rrf-1(pk1417))* strain	tissue-spec.	L1	48h post L1; (pre-reproductive)
Fig. S3	e-h	body size of *rab-1* RNAi exposured *lag-2* promoter specific RNAi (*lag-2p::mNG::PH::F2A::rde-1)* strain	tissue-spec.	L1	48h post L1; (pre-reproductive)
Fig. 3	a-c	*lag-2* promoter specific RNAi (*lag-2p::mNG::PH::F2A::rde-1)* on *rab-1* RNAi in pre-reproductive animals	tissue-spec.	L1	48h post L1; (pre-reproductive)
Fig. 3	d-f	Uterine-specific RNAi (*fos-1a::rde-1)* on *rab-1* RNAi	tissue-spec.	L1	72h post L1; (controls are young adults)
Fig. 4	a-f	*lag-2* promoter specific RNAi (*lag-2p::mNG::PH::F2A::rde-1)* on *rab-1* RNAi in reproductive adults	tissue-spec.	L1	72h post L1; (controls are young adults)
Fig. S4	a-g	Locations of lag-2p::mNG::F2A::rde-1 rescue transgene expression during development	NA	NA	see detailed stage labeling in figure panels (figure S4)
Fig. 5	a-f	DAPI stain of *lag-2* promoter specific RNAi (*lag-2p::mNG::PH::F2A::rde-1)* on *rab-1* RNAi in reproductive adults	tissue-spec.	L1	72h post L1; (controls are young adults)
Fig. 5	g-i	DAPI stain of *lag-2* promoter specific RNAi (*lag-2p::mNG::PH::F2A::rde-1)* on *rab-1* RNAi in reproductive adults	tissue-spec.	L1	96h post L1; (controls are past young adults)

Table S2. RNAi clones made for this study

C. elegans rab gene	Forward Primer	Reverse Primer	RNAi insert length*
glo-1	5'-CGTATTTGCCAAAAATTTGAACGCC-3'	5'-AGGCCAAGACCGATATGGGG-3'	364 bp
Y71H2AM.12	5'-GAACTCACTCACTAACAAGATCGTGC-3'	5'-GTCAATTCGCCTTCAATTATGGG-3'	276 bp
rab-14	5'-CGTATTTGGATTGGTCAAGC-3'	5'-AAATGACGGCTGCTCCTTAC-3'	499 bp

Table S3. List of all strains used in this study

**Strain**	**Genotype**	**Source**
N2	wildtype	CGC
KLG019	*naSi2(mex-5p::H2B::mCherry::nos-2 3'UTR) II; inx-9(qy79(GFP::inx-9)) IV.*	Gordon et al., 2020
NK2571	*qy78[mKate::inx-8] IV; cpIs122[lag-2p::mNeonGreen:: PLC$^{\delta PH}$] II.*	Gordon et al., 2020
MAH23	*rrf-1(pk1417) I.*	Kumsta and Hansen, 2012
NK2115	*cpIs121[lag-2p::mNG::PH::F2A::rde-1] I; rrf-3(pk1426) II; rde-1(ne219) V.*	Linden et al., 2017
NK1316	*qyIs102[fos-1ap::rde-1]; rrf-3(pk1426) II; qyIs10[lam-1p::lam-1::GFP] IV; rde-1(ne219) V; qyIs24[cdh-3p::mCherry::PLCPH] X.*	Morrisey et al., 2014
